# Supplementary material for: The interactome of intact mitochondria by cross-linking mass spectrometry provides evidence for coexisting respiratory supercomplexes
Source: Mol Cell Proteomics. 2017 Dec 8;17(2):216–32. doi: 10.1074/mcp.RA117.000470 (PMC5795388; doi:10.1074/mcp.RA117.000470)
Supplement: Supplemental Data [file supp_RA117.000470_133922_0_supp_23978_fzffwf.zip › spectra_annotation/mito_spectra_annotation/166-1-10-1-5-1.pdf]

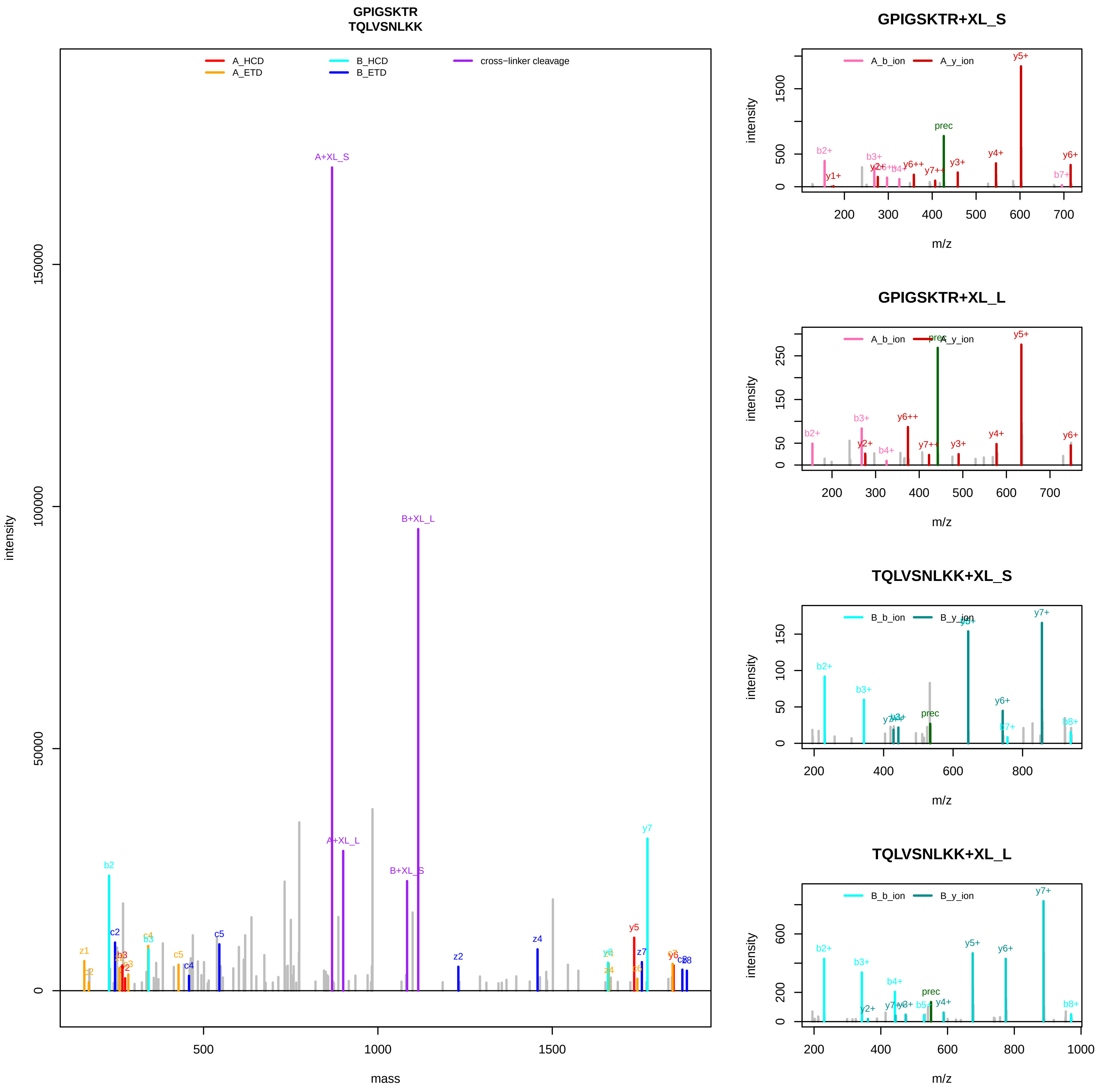
GPIGSKTR
TQLVSNLKK
A_HCD
A_ETD
B_HCD
B_ETD
cross−linker cleavage
A+XL_S
B+XL_L
A+XL_L
B+XL_S
intensity
mass
0
50000
100000
150000
500
1000
1500
b2
c2
c4
b3
c5
c4
z1
c2
b3
y2
c3
c5
z2
z4
y7
y5
z7
c6
y6
GPIGSKTR+XL_S
GPIGSKTR+XL_L
TQLVSNLKK+XL_S
TQLVSNLKK+XL_L
A_b_ion
A_y_ion
B_b_ion
B_y_ion
prec
m/z
y5+
y4+
y6+
y3+
y6++
y7++
y2+
b2+
b3+
b4+
b7+
y1+
y7+
y6+
b8+
b5+
b7+
y2+
y3+
y4+
y5+
200
300
400
500
600
700
800
1000
